# Philadelphia Lying-In Charity

**Published:** 1887-08

**Authors:** Harry Huzza

**Affiliations:** Rome, Ga.


### THE PHILADELPHIA LYING-IN CHARITY.

*Editors Atlanta Medical and Surgical Journal:*

The question of obstetrics is always a perennial though often a prosy subject. Being as old as Eden, there is little of novelty, however much there should be of interest to all of us. It is a department of medicine which is of great, if not greatest, importance to the young practitioner. In no other class of patients can he give rise to such an amount of criticism, whether favorable or unfavorable, and nowhere, than in the lying-in chamber, does he require more tact, self-possession and knowledge in order to subserve the best interests of his patients, himself and the general public. Leaving to older heads the "interesting" questions of midwifery, it is still worth one's while to inquire "how and where this knowledge, most useful to the physician, may be obobtained?" He reads his text-books as a student; he listens to learned expositions; but no didactic lecture can impart that skill and confidence, and that *tactus eruditus* which experience alone cultivates, if it does not furnish. This is all gained in time by the regular physician after many blunders, and paid for with tribulation of mind and often grievous mistakes. How this knowledge can be systematically learned in Paris and Munich has been duly set forth in some late journals (New York *Medical Journal*, May 28th, 1887, pp. 598, 600). I propose to give a short description of the Philadelphia Lying-in Charity, and describe one of the places where such instruction can be obtained in this country.

This institution is largely the result of the mind and labors of one man, Dr. Joseph Warrington. He organized it in June, 1837, "for the practical training of physicians and nurses in their duties to pregnant, parturient and puerperal women." It was based upon and included the obstetrical department of the Philadelphia Dispensary, founded in 1786; the Philadelphia Lying-in Charity, incorporated in 1832; the Philadelphia Nurse Society, established in 1839. Its success and efficiency were due to the indefatigable and arduous efforts of Dr. Warrington, who, as long as he lived, preserved the high standard in teaching on which the institution was founded. It was one of the first, if not the first, institution of its kind, and certainly the first to systematically train nurses and send them out as aids to the doctors and the community. But leaving out its history and growth, I purpose to give a short description of the hospital as it is, of its methods, teachings and benefits.

At the opening of the fall term of the medical colleges, the physicians in charge organize their class and begin their regular course in obstetrics. This includes forty lectures and six months' practical work. Besides this, there are short courses of six weeks each, embracing only bed-side instruction in the hospital. An extract from the rules will explain the attitude of the institution to its students: "The pupil physicians, being appointed from the medical class and comprising any or all of its members, are expected to feel their full accountability to the managers. They shall accept cases assigned them as involving the usual responsibilities and duties of regular professional engagements, to be treated with the same regard for their safety, and the same deference to their feelings as would be expected from the humane and conscientious physician toward his private patients." The idea underlying all their methods and all their teachings, even in their in-door work, is to treat patients as nearly as possible as a physician would his own private cases. This is a very important point to consider in connection with this subject. Its influence is very wide-reaching. The German methods, the English methods, the methods of all public lying-in hospitals include means and ends which are expensive, cumbersome and unnecessary, and which, while suitable for public charities, will not be tolerated in private practice. This point of view is too much neglected in medical teaching. General instruction, to be effective, must be practical, able to be carried out anywhere within reasonable limits.

The patients who apply to the Philadelphia Lying-in Charity are divided into two classes: First, those who are too poor to employ a physician, but who have homes, however humble these may be. The woman's address is taken, together with certain details of the case, and she is assigned to one of the pupil physicians who calls upon her at once. She is afterwards treated just as any private patient, except that her name is furnished to the lady visitor of the district, who sees that any want or need that may require charitable assistance is relieved.

Second. Cases of emergency, and those so poor they have no place to stay, are taken into the obstetric wards of the hospital. When labor begins in any of these in-door cases, as soon as the preliminary pains come on, the patient is put to bed and a vaginal injection of mercuric bichloride solution, 1 to 1,000, is given. The bowels are cleared with a copious enema. The resident physician, a female graduate, takes charge of the case, sends for the pupil physicians on the list, which always includes the new men and the less experienced. The bed is properly made up and all details attended to by the nurses of the training-school. The physician in charge, who had been sent for the first thing, soon arrives and takes charge of the case. He usually gives clinical bedside instruction. The hands of all who examine the patient are thoroughly cleansed with soap and water, with nail-brush also, and disinfected in solution of 1 to 1,000 mercuric bichloride. Carbolized oil is used for lubricating the fingers. The patient is always treated with the utmost consideration. Only a few of the pupil physicians are present at any one case. The examinations are few, and limited to one or two at a time with long intervals. As much as possible the lessons and procedures taught in the lecture-room are illustrated in the progress of the parturition. Especial attention is paid to preserving the perineum, delivering the placenta and management of the new-born child. Of course, when needed, the forceps are applied, but the teaching on this point is very conservative. After delivery the dressing is finished by the application of a binder and an antiseptic pad over the vulva, simply a roll of wood-wool folded diagonally in a square of dry antiseptic gauze and pinned to the binder, front and back. A chart is hung beside each bed on which the particulars of position and presentation, pulse, respiration and temperature, morning and evening, are recorded, together with any details of treatment. When the patient is discharged, this chart is filed away and preserved. A pupil physician is expected to witness three such cases indoors before being sent to outdoor cases. I should add that only graduates and advanced students, who are attending at least their second course of lectures, are admitted to these classes.

The pupil physicians are thus instructed in the management of cases, not only at the bedside, but in the regular lectures, which are made specially practical, dealing with main outlines and including careful training in manipulation with a Budiss manikin. When they are sent to out-door cases, everything possible is done to make them appreciate the dignity and importance of their duties. In case of the least doubt or perplexity, they can call on either of the principal physicians or their assistants. When there are complications they are required to do so, and forceps are never to be applied except by the principal.

Of course this is only a hasty outline of the field covered by this institution, but a little reflection will serve to show how useful and unique it is. As an example of their results, in the winter of 1885 they reached 1,000 consecutive deliveries, including in-door and out-door cases, without losing a single mother, and only two babies died—a record almost unparalleled this certainly is. Moreover, by the statistical table of the "death-rate of lying-in hospitals in the United States" (*Medical News*, March 5th, 1887, p. 253), in-door cases only, it is shown that the Philadelphia Lying-in Charity ranks third in point of excellence, the death-rate being but 0.57 per cent., or to put it plainly, about ½ of 1 per cent., or one woman in 200 dies.

One might speak also of the gynæcological department, including two large wards and private single rooms; of the daily clinic for women and tri-weekly for children; of the nurse-training school, home and directory, the oldest in this country and the first ever organized in the world; and above all, of the organized charity of the lady visitors who look after all these patients and distribute charities where they are needed.

I will have accomplished my object if I have made clear that there is an institution where such thorough, systematic instruction in obstetrics is given, at the same time preserving all the relations and modes of procedure adapted to private patients. It is this feature which renders the instruction so valuable and practically useful. At another time I shall endeavor to give you some results and cases which will illustrate still further the methods and attainments under this institution's teachings.

Yours truly, HARRY HUZZA, M. D.

*Rome, Ga., July 1st, 1887.*

